# Resolving the renal microenvironment: a 5-plex immunofluorescence workflow to quantify B-lineage cells in FFPE lupus nephritis biopsies

**DOI:** 10.3389/fimmu.2026.1774536

**Published:** 2026-03-25

**Authors:** Caleb Chan, Patrick S. Chang, Sreedevi Chalasani, Janet Tao, Harini Raghu, Cary D. Austin

**Affiliations:** 1 Department of Pathology, Genentech, A Member of the Roche Group, South San Francisco, CA, United States; 2 Department of Translational Medicine, Genentech, A Member of the Roche Group, South San Francisco, CA, United States

**Keywords:** antibody-secreting cells, ASCs, B cells, FFPE, immunofluorescence, lupus nephritis, multiplex, plasma cells

## Abstract

Accurate identification and spatial enumeration of B lineage cells in formalin-fixed, paraffin-embedded (FFPE) lupus nephritis (LN) kidney tissue is critical for understanding disease pathogenesis and CD20-directed therapeutic responses. We developed a targeted 5-plex immunofluorescence and digital image analysis workflow for simultaneous enumeration of B cells, plasma cells (PCs), and plasmablasts (PBs) in FFPE human tissues that is amenable to deployment in LN clinical trials. We validated this workflow on two tonsils and eight LN biopsy tissue blocks. Comparison of B lineage markers confirmed that CD79a provides far superior sensitivity for interstitial B cells compared to CD19, establishing it as the requisite anchor for B cell burden assessment in FFPE lupus nephritis tissue. Accordingly, B cells were defined as CD79a^+^/CD138^-^; PCs as CD138^+^/CD38^+^/Ki-67^-^; and PBs as CD138^+^/CD38^+^/Ki-67^+^. These definitions ensured unambiguous cell classifications, overcoming the challenge of variable CD138 expression in renal tubular epithelium. Whole slide analysis of LN tissues revealed comparable average frequencies of B cells versus PCs (~250 cells/mm^2^), with far fewer PBs (~14 cells/mm^2^). Comparison with an exploratory “permissive” gating strategy (CD38^+^/Ki-67^+^) confirmed the absence of CD138^-^ B-lineage plasmablasts, validating the sufficiency of CD138 for tissue ASC enumeration. Most PCs and PBs were CD79a^+^, indicating the retention of a functional B-cell program that may contribute to disease pathogenesis. This robust, validated, fit-for-purpose methodology is poised for deployment in larger clinical LN cohorts to evaluate local tissue impacts of B-cell depletion therapies, deepening our understanding of disease pathogenesis and treatment responses.

## Introduction

Systemic Lupus Erythematosus (SLE) is a debilitating autoimmune disease characterized by chronic inflammation and tissue damage, with lupus nephritis (LN) being one of its most severe manifestations (1). A core feature of SLE pathology is the breakdown of B cell tolerance, leading to the sustained production of pathogenic autoantibodies and the differentiation of B cells into antibody-secreting cells (ASCs), namely plasmablasts (PBs) and plasma cells (PCs), which mediate kidney injury. Given their central role in driving pathogenesis, these B lineage cells are key therapeutic targets. Recent high-dimensional spatial analyses have reinforced this importance, demonstrating that specific *in situ* B cell neighborhoods are strongly associated with clinical outcomes (2). These findings highlight the critical need to accurately monitor these populations within the kidney tissue, particularly to evaluate the pharmacodynamic efficacy of B cell-directed therapies in interventional clinical trials.

Despite the clear involvement of these B lineage populations in LN immunopathology, accurate quantification in routine formalin-fixed, paraffin-embedded (FFPE) specimens presents specific challenges for monitoring therapeutic response. Biologically, B cell activation drives differentiation into proliferative plasmablasts (Ki-67^+^) and ultimately into quiescent, post-mitotic plasma cells (Ki-67^-^) (3, 4). This maturation process is characterized by the downregulation of pan-B cell markers and the upregulation of ASC markers such as CD38 and CD138. Conventional immunohistochemical (IHC) approaches in clinical trial tissue often rely on single markers such as CD20, which fail to capture the full spectrum of differentiation, as ASCs downregulate CD20 during maturation (3). Furthermore, CD20 expression can be masked or internalized during therapeutic depletion, rendering it an unreliable metric for residual tissue cellular burden in treated patients (5). In addition, reliance on CD38 alone is insufficient due to its expression on other lymphocyte populations, including NK cells and activated T cells. While assessing multiple markers could resolve these phenotypic overlaps, reliance on serial sections is often unfeasible in LN clinical trials, where needle biopsy material is scarce and unstained slides for exploratory analysis are limited. Finally, the renal microenvironment presents additional unique obstacles; specifically, the often underappreciated endogenous expression of CD138 (Syndecan-1) on renal tubular epithelium (6, 7) can confound the identification of CD138^+^ PCs, creating a significant risk of false positives.

To address these specific challenges, we developed a targeted 5-plex immunofluorescence (IF) and digital image analysis workflow for the simultaneous enumeration of B cells, PCs, and PBs in FFPE human tissues. We anchored our B-lineage detection on CD79a, a robust marker utilized in diagnostic pathology to identify B cells across diverse tissues, including lymphoid organs and solid tumors (8, 9). CD79a remains detectable even when CD20 is lost or masked following antibody therapy (10, 11). We therefore multiplexed CD79a with CD19, CD138, CD38, and Ki-67 to establish a rigorous cell immunolabeling and classification strategy. The inclusion of CD19 allowed us to benchmark this canonical flow cytometric marker against CD79a to determine the optimal immunolabeling strategy for the FFPE renal microenvironment. This approach clearly discerns B-lineage populations from each other while overcoming problematic renal tubular interference. Here, we present the validation of this workflow in tonsil (TN) and lupus nephritis biopsy tissues, demonstrating its utility as a high-precision tool for evaluating B cell dynamics and longitudinal therapeutic responses in clinical cohorts.

## Materials and methods

### Tissue

Archival FFPE human tissues were acquired from Avaden Biosciences under warranty that appropriate IRB approval and informed consent were obtained. Tissues used for initial assay development included two tonsils and two normal kidney tissues. Tissues used for assay deployment and quantitative analysis included eight LN biopsy blocks and two human tonsils. The LN cohort (**Supplementary Table 1**) comprised four female and four male donors (median age 40.5; range 21–67). The specimens were collected between 1984 and 2012 and were accompanied by clinical diagnoses that the vendor mapped to the ISN/RPS 2003/2018 classification system (10). These cases represent a spectrum of proliferative disease, including Class III (n=1), Class III+V (n=1), Class IV (n=4), and Class IV+V (n=2). Initial clinical diagnoses provided by the vendor were independently verified via internal histopathological review of H&Es by an anatomic pathologist (C.D.A.; **Supplementary Figure 1**).

### Immunohistochemistry and immunofluorescence

All immunolabeling was performed on 4 µm sections of FFPE human tissue mounted on glass slides using a Ventana Discovery Ultra autostainer (Roche Diagnostics, Tucson, AZ). Single label chromogenic immunohistochemistry was performed using Cell Conditioning 1 (CC1, #950-500) antigen retrieval and primary antibody incubation with anti-CD19 (1:100/0.83 µg/ml, clone LE-CD19, #M7296, Agilent, Santa Clara, CA). Detection was performed using the OmniMap anti-Mouse HRP system (#760-4310) followed by ChromoMap DAB visualization (#760-159).

A 5-plex immunofluorescence assay was performed using a Tyramide Signal Amplification (TSA)-based sequential multiplexing strategy (12). Unstained slides were baked for 30 min at 70°C, followed by a single initial antigen retrieval step using CC1 for 64 min at 100°C. This technique employs iterative cycles of single-marker immunolabeling; a blocking step in DISCOVERY Inhibitor (Neutralize) reagent (#760-4840) was performed before every staining round for 8 min at room temperature (RT). Each immunolabeling cycle consisted of primary antibody incubation, secondary detection, and tyramide signal deposition, followed by heat elution in Cell Conditioning 2 (CC2) buffer (#950-223) for 8 min at 100°C to remove the antibody complex before the next cycle, while the fluorophore tyramide deposits remained covalently bound to the tissue. We determined epitope tolerance to iterative heat-induced epitope retrieval cycles to inform the final sequence position assignments (**Table 1**). Primary antibody concentrations, TSA incubation times, and fluorophore assignments were empirically optimized for each channel to maximize the signal-to-noise ratio and minimize spectral spillover.

**Table 1:** epitope tolerance for sequential multiplexing order.

% change in mean fluorescence intensity relative to position 1							
Marker	Clone	Position 1 (no elutions)	Position 2 (1 elutions)	Position 3 (2 elutions)	Position 4 (3 elutions)	Position 5 (4 elutions)	Final assignment
CD79a	SP18	0	30.64	11.70	10.38	12.78	Position 2
CD38	SPC32	0	-1.85	-4.10	-19.20	-15.88	Position 1
Ki-67	D3B5	0	14.84	38.94	38.57	5.31	Position 4
CD138	B-A38	0	40.83	42.98	24.52	13.10	Position 5
CD19	LE-CD19	0	-21.57	278.06	163.59	197.18	Position 3

Single-plex immunolabeling on two FFPE TN sections was performed using the TSA-based IF protocol of the 5-plex assay, preceded by 0–4 iterative elution cycles to simulate the 5-plex sequence positions. Thresholded mean fluorescence intensity (MFI) was quantified, and the percent change in MFI relative to the non-eluted control (Position 1) is tabulated. The final marker sequence assignment for the 5-plex panel is reported in the ‘Final Assignment’ column”.

The final optimized 5-plex IF assay was sequenced as follows: anti-CD38 clone SPC32 (#NCL-L-CD38-290, Leica Biosystems, Deer Park, IL) with anti-mouse OmniMap-HRP (#760-4310) detection and TSA-Rhodamine 6G (#760-244) visualization; anti-CD79a clone SP18 (#790-4432, Roche Diagnostics) with anti-rabbit OmniMap-HRP (#760-4311) detection and TSA-FAM (#760-243) visualization; anti-CD19 clone LE-CD19 with anti-mouse OmniMap-HRP detection and TSA-Cy5 (#760-238) visualization; anti-Ki-67 clone D3B5 (#12202, Cell Signaling Technology, Danvers, MA) with anti-rabbit OmniMap-HRP detection and TSA-Red 610 (#760-245) visualization; and anti-CD138 clone B-A38 (#760-4248, Roche Diagnostics) with anti-mouse OmniMap-HRP detection, followed by signal amplification using TSA-DIG and visualization with Opal Polaris 780 (anti-DIG) (#FP1501001KT, Akoya Biosciences, Marlborough, MA). Full assay parameters, including antibody concentrations and specific incubation conditions, are detailed in **Supplementary Table 2**. Nuclei were counterstained with DAPI (#D1306, Thermo Fisher Scientific, Waltham, MA). Human tonsil tissue served as the biological positive control to confirm appropriate subcellular localization and signal specificity for all markers. Isotype control immunolabeling confirmed the absence of non-specific primary antibody binding. ‘Leave-one-out’ negative controls, in which one antibody at a time was replaced with diluent in the 5-plex assay, confirmed the lack of significant cross-reactivity or optical bleed-through between detection channels.

### Image acquisition

Digital whole-slide images were acquired with a 20x objective lens at a resolution of 0.325 µm/pixel using an Olympus VS200 slide scanner equipped with specialized filter cubes optimized for Roche/Ventana fluorophores. Seven distinct fluorescence channels were captured: DAPI (nuclei), CFP (autofluorescence/background), FITC (CD79a), Rhodamine 6G (CD38), Red 610 (Ki-67), Cy5 (CD19), and CY7 (CD138).

### Digital image analysis

Whole slide image analysis was performed to quantify cell phenotypes and spatial densities within the analyzable tissue area. First, tissue detection and total tissue area calculations were performed using a custom Python script by applying Gaussian blur and thresholding on the autofluorescence channel of the whole slide images. Analysis regions of interest were refined by manual and automated exclusion of non-tissue whitespace, staining artifacts, and non-renal tissue. Single-cell segmentation was performed using InstanSeg (version 0.0.9, Pytorch package) utilizing the fluorescence_nuclei_and_cells pre-trained model. Following segmentation, mean fluorescent intensity was extracted for each cell across all channels using custom scripts written in Python (version 3.10). Cell phenotypes were defined by determining marker positivity via intensity thresholding. Cutoff values for each marker were set independently and optimized using custom Python scripts utilizing Matplotlib widgets for interactive evaluation to ensure accuracy. To minimize false positives, cells exhibiting high signal in the autofluorescence (CFP) channel were systematically rejected. Finally, positive cell counts were integrated with the calculated tissue areas to generate cell densities. Cell classifications based on marker status were defined as follows: B cells (CD79a^+^/CD138^-^), PCs (CD138^+^/CD38^+^/Ki-67^-^), ‘permissive’ PBs (CD38^+^/Ki-67^+^), bona fide PBs (CD138^+^/CD38^+^/Ki-67^+^). Importantly, these definitions were permissive regarding markers not explicitly used for exclusion; for example, the B cell class (CD79a^+^/CD138^-^) included all lineage-positive cells regardless of variable expression of CD38 or Ki-67, ensuring capture of activated or proliferating subsets. Each classified cell population was enumerated individually per slide and cumulatively across all slides of each tissue type. Data were reported as: i) cells per mm2 of tissue, and ii) cells per total number of tissue cells. Additionally, the proportions of CD79a^+^ and CD19^+^ subpopulations comprising each cell class were calculated, as well as the proportion of bona fide plasmablasts relative to total antibody-secreting cells (defined as bona fide PBs + PCs). Evaluation of ‘leave-one-out’ control slides confirmed that spectral bleed through/crosstalk was negligible, as the thresholding used for cell classification successfully excluded these faint signals. Selected data were tabulated then plotted using GraphPad Prism 10 (GraphPad Software, Boston, MA).

### Ethics statement

The human tissues used in this study (tonsil and lupus nephritis kidney) were de-identified, archival specimens acquired from Avaden Biosciences. These specimens consist of remnant clinical tissue procured from U.S.-based hospitals in full compliance with HIPAA, IRB protocols, and The Common Rule, with written informed consent for research use obtained by the vendor at the time of collection. As the research involved exclusively the secondary use of de-identified specimens, the study was determined to be exempt from independent IRB review in accordance with federal and institutional requirements.

## Results

### Assay design and marker assessment

**Figure 1:**
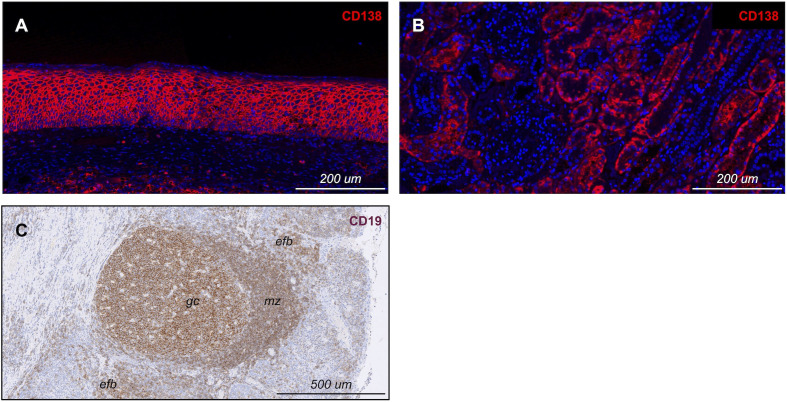
Assessment of potential confounding immunolabeling patterns. **(A, B)** Anti-CD138 immunoreactivity on tonsillar **(A)** and renal tubular **(B)** epithelium (identified by its characteristic morphology of cuboidal cells enclosing a central lumen), reflecting endogenous CD138 expression that interferes with definitive PC and PB identification. **(C)** Anti-CD19 immunolabels tonsillar B cell follicles, with robust labeling of germinal centers (gc), but weaker labeling in the mantle zone (mz) and extrafollicular B cells (efb) by IHC.

To implement the strategy outlined above, we constructed a multiplex IF panel designed to discern B cells, PCs, and PBs from each other and from non-B-lineage cells, such as other inflammatory cells and renal epithelium. During the initial optimization of the PC marker CD138, we observed prominent, unexpected immunoreactivity on the renal tubular epithelium, identifiable as organized structures composed of cuboidal cells lining distinct lumens (**Figures 1A, B**). While this pattern initially raised concerns regarding antibody specificity, a comparison with reference immunolabeling patterns in the Human Protein Atlas (13) and review of the CD138 expression literature (6, 7) confirmed that the renal tubular immunolabeling reflected true endogenous CD138 expression. This confounding expression rendered CD138 alone insufficient for specific PC identification in the kidney. To resolve this, we incorporated CD38 as an obligate co-marker, as it is highly expressed on both PCs and PBs (14, 15). While CD38 is also expressed on other lymphocyte subsets (e.g., NK cells, activated T cells), these populations lack CD138; thus, this dual-positive requirement (CD138^+^/CD38^+^) effectively distinguished true antibody-secreting cells (ASCs) from both CD138^-^ lymphocytes and CD138^+^/CD38^-^ tubular epithelium.

**Figure 2:**
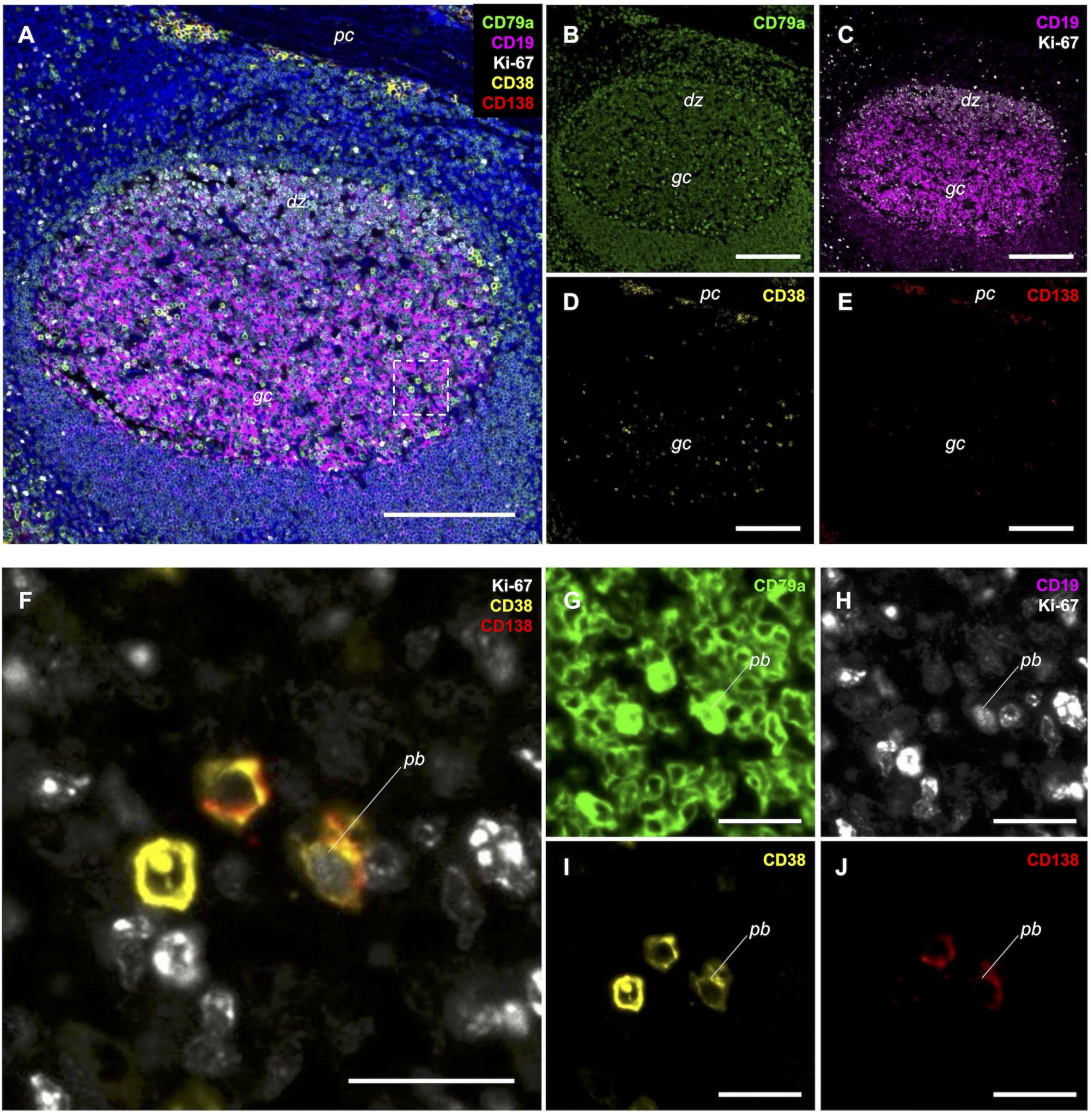
5-plex immunofluorescence immunolabeling on FFPE tonsil sections confirms expected expression patterns. Low magnification views **(A–E)** and high magnification views **(F–J)**; inset in A indicates the germinal center region shown in high-magnification panels **(F–J)**. CD79a [green, **(A, B, G)**] immunolabels B lineage cells clustered within follicles and scattered throughout extrafollicular regions. CD19 [magenta, **(A, C)**] robustly immunolabels germinal center (gc) B cells, with other populations labeling weakly or not at all. Ki-67 [white, **(A, C, F, H)**] immunolabels nuclei of actively dividing cells, including centroblasts in the dark zone (dz) and a rare plasmablast (pb) **(F–J)**. CD38 [yellow, **(A, D, F, I)**] immunolabels plasma cells (pc) and high-expressing activated B cells. CD138 [red, **(A, E, F, J)**] immunolabels plasma cells and plasmablasts. **(A)** is a 5-marker composite; **(B–E)** show single or dual markers. **(F)** is a composite of Ki-67, CD38, and CD138 highlighting a rare bona fide plasmablast (pb) within a germinal center, while panels **(G–J)** show the corresponding single markers (including CD79a). Scale bars = 200 µm **(A–E)** and 25 µm **(F–J)**. Composite **(A)** includes DAPI (blue) to visualize the tonsillar architecture. Note: Display settings were optimized to visualize lower-intensity populations, resulting in apparent visual saturation of the brightest cells; quantitative analysis was performed on raw data within the linear dynamic range.

We concurrently evaluated the performance of CD19 as a secondary B cell marker. While CD19 is the established gold standard for evaluating peripheral B cell depletion by flow cytometry (16), its detection in FFPE tissue is often compromised by epitope instability. Nevertheless, a prior study successfully used single label CD19 IHC to demonstrate B cell depletion following CD19-targeted therapies in solid tissues (17). As CD19 IHC is neither widely used nor standardized, we employed the same antibody clone (mAb LE-CD19) utilized in that work. Notably, while this clone has been sufficient to visualize depletion in lymph nodal tissue, comparison with matched CD20 immunolabeling in that publication suggests that CD19 immunoreactivity is intrinsically less robust than that of CD20. Consistent with this, while our single-label IHC assay using this clone yielded results in lymphoid tissue comparable to those previously published (**Figure 1C**), we observed marginal sensitivity on B cells outside of germinal centers (GCs). This limitation became more pronounced in the multiplex IF format: in tonsil controls, immunolabeling was bright in GCs but exhibited insufficient signal intensity in other B cell populations to permit reliable intensity thresholding (**Figure 2**). In contrast, CD79a robustly and uniformly labeled the B-lineage compartment, clearly identifying B cells clustered within follicles and loosely scattered throughout extrafollicular regions. This direct comparison confirmed that CD79a offered superior, reliable signal intensity across the B cell populations compared to CD19, reinforcing the necessity of our CD79a-anchored approach for comprehensive B cell enumeration in FFPE specimens.

### Multiplex optimization and signal specificity

**Figure 3:**
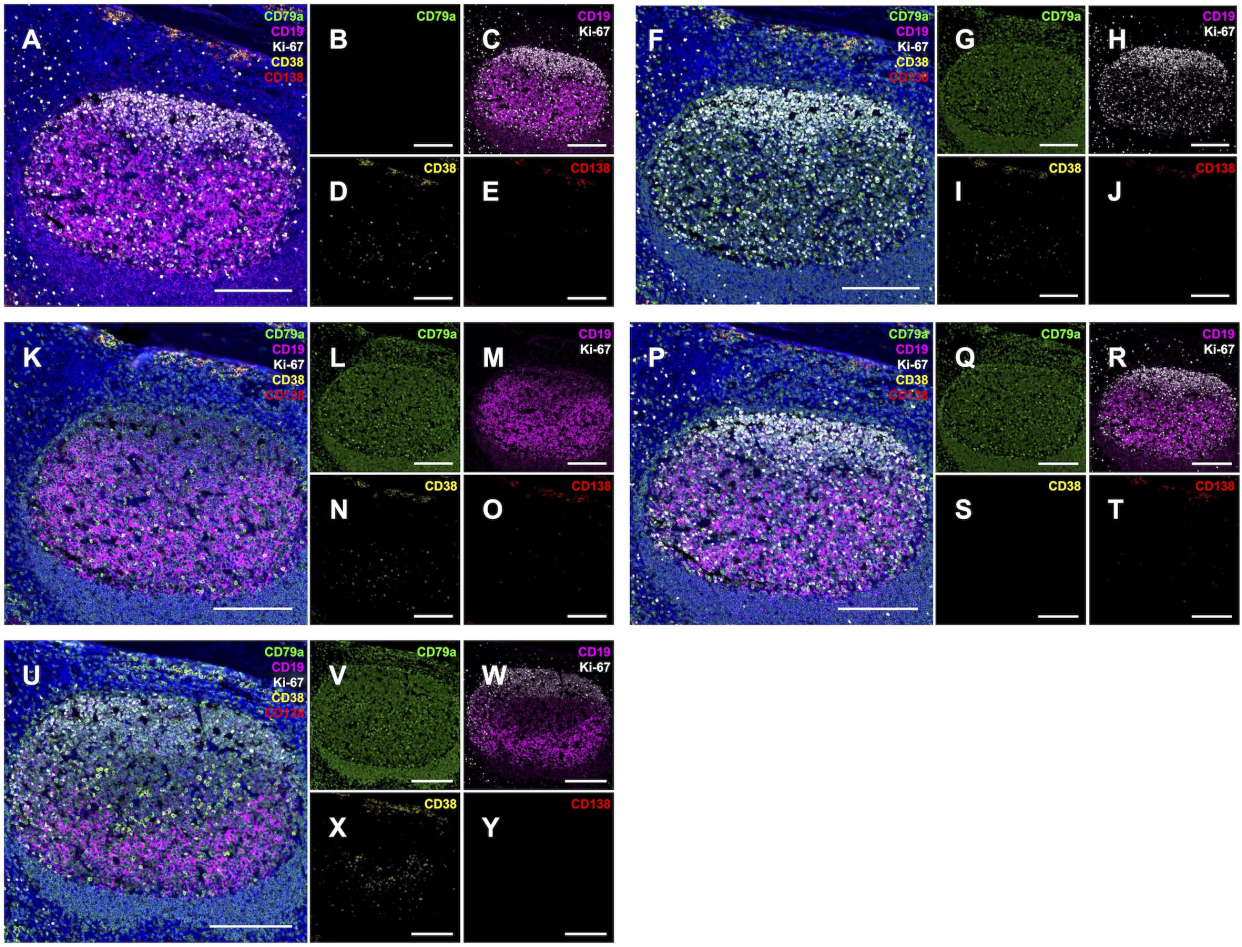
‘Leave-One-Out’ 5-plex immunofluorescence control immunolabeling. Performed on semi-serial FFPE tonsil sections to confirm absence of channel-to-channel interference from optical bleedthrough or secondary detection crosstalk. Five control slides are shown, each with one primary antibody omitted: anti-CD79a **(A–E)**, anti-CD19 **(F–J)**, anti-Ki-67 **(K–O)**, anti-CD38 **(P–T)**, and anti-CD138 **(U–Y)**. Detection channels include: CD79a [green, **(A, B, F, G, K, L, P, Q, U, V)**], CD19 (magenta, **(A, C, F, H, K, M, P, R, U, W)**), Ki-67 [white, **(A, C, F, H, K, M, P, R, U, W)**], CD38 [yellow, **(A, D, F, I, K, N, P, S, U, X)**], and CD138 [red, **(A, E, F, J, K, O, P, T, U, Y)**]. Larger panels **(A, F, K, P, U)** are 5-plex composites including DAPI (blue); small panels show single channels except for dual CD19/Ki-67 displays in **(C, H, M, R, W)**. Scale bars = 200 µm.

Given that our primary antibodies were derived from mouse or rabbit hosts, we utilized a tyramide signal amplification (TSA)-based sequential multiplexing strategy. To confirm signal specificity, the assay was evaluated in tonsillar (TN) control tissue, confirming that immunoreactivity patterns matched established lymphoid architecture (18) (**Figure 2**). Next, we confirmed the specificity of the PC and PB markers by verifying their restriction to established anatomical niches. CD38 and CD138 showed co-localization on large, strongly immunoreactive extrafollicular PCs, predominantly localized to subepithelial, peri-cryptal, and deep connective tissue septal zones as previously described (18). This distinct dual-positive phenotype allowed for clear discrimination from CD38^+^ single-positive activated B cells within the GC and CD138^+^ single-positive surface and crypt epithelium. Ki-67 immunolabeling highlighted the proliferative compartment, including scattered cells within and outside of GCs and dense clusters of centroblasts within GC dark zones. We also observed rare bona fide PBs (CD38^+^/CD138^+^/Ki-67^+)^ in the germinal centers, perifollicular, and parafollicular regions (**Figures 2F–J**). To assess potential interference from secondary detection crosstalk, a panel of 5-plex ‘Leave-One-Out’ (LOO) negative control slides was prepared and imaged identically to the full assay. Analysis of these controls confirmed no discernible signal from nonspecific artifacts in any channel (**Figure 3**). Collectively, these data demonstrate the panel’s specificity and its capacity to distinguish true B-lineage targets from CD138^+^ epithelial immunolabeling within the tonsillar tissue architecture.

### Assay deployment and image analysis

**Figure 4:**
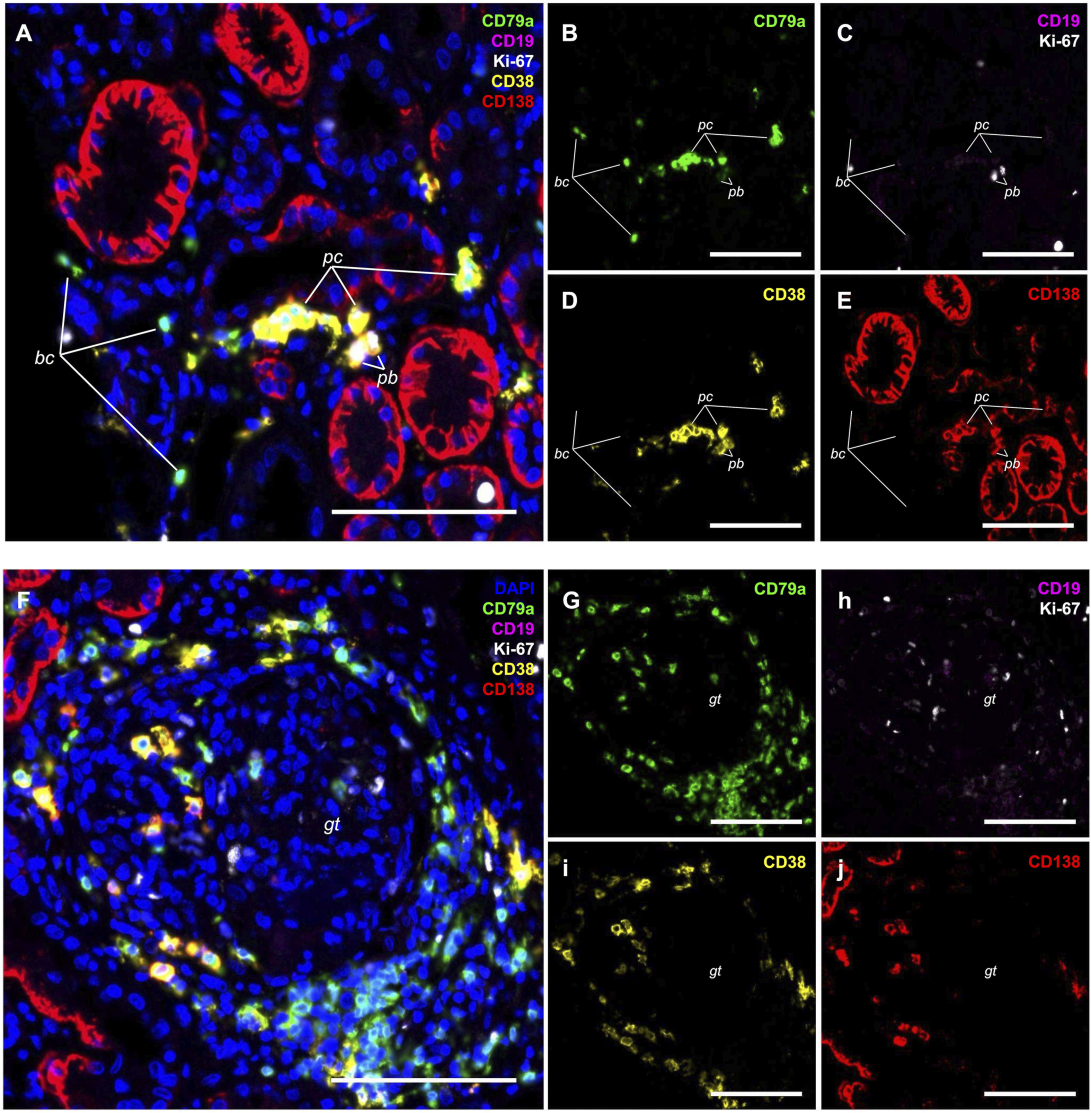
5-plex immunofluorescence immunolabeling on FFPE lupus nephritis sections. Representative images from the cohort are shown (see **Supplementary Figures 2** and **3** for intermediate- and low-magnification overviews of the full biopsy cohort). The assay identifies infiltrating tubulointerstitial B lineage cells: CD79a^+^/CD138^-^ B cells (bc), CD138^+^/CD38^+^/Ki-67^-^ plasma cells (pc), and rare CD138^+^/CD38^+^/Ki-67^+^ plasmablasts (pb). **(A–E)** Representative interstitial infiltrate; renal tubular cells variably express CD138 but are CD38^-^. Markers: CD79a (green, A and B), dual CD19 (magenta) and Ki-67 (white) **(A, C)**, CD38 [yellow, **(A, D)**], and CD138 (red, **(A, E)**). **(F–J)** Periglomerular infiltrate with markers corresponding to **(A–E)**: CD79a [green, **(F, G)**], dual CD19 (magenta) and Ki-67 (white) **(F, H)**, CD38 [yellow, **(F, I)**], and CD138 (red, **(F, J)**). Composites **(A, F)** include DAPI (blue) to visualize the renal architecture, including a glomerular tuft (gt) in F. Note the cuff of periglomerular B cells and PCs, with scattered cells infiltrating the glomerular tuft itself. Single-channel panels are provided to resolve specific immunolabeling patterns and distinguish true co-localization from additive color mixing. Scale bars = 100 µm.

The 5-plex assay was deployed on a panel of eight LN needle biopsy tissue blocks and two TN. Visual inspection across the cohort confirmed robust and consistent staining performance and signal integrity despite tissue heterogeneity (**Supplementary Figure 2**). Infiltrating B cells (CD79a^+^/CD138^-^), PCs (CD138^+^/CD38^+^/Ki-67^-^), and rare bona fide PBs (CD138^+^/CD38^+^/Ki-67^+^) were identified within the LN tubulointerstitium and were heterogeneously distributed (**Figures 4A–E**). B cells and ASCs, when present, were frequently distributed as diffusely scattered individual cells throughout the tubulointerstitium. However, in tissues with higher infiltrate density, we observed focal aggregates of variable size and density, often partially intermingled and situated in perivascular and periglomerular regions, with the latter occasionally accompanied by individual infiltrative cells scattered within the glomeruli themselves (**Figures 4F–J**). Notably, we observed that ASC density often paralleled B cell density across the cohort of tissues and even within infiltrated tissue regions; tissues and regions with sparse B cell infiltrates generally showed sparse ASC presence, while those with dense B cell aggregates exhibited robust ASC accumulation.

LN B cells often lacked discernible CD19 immunoreactivity. LN PCs and bona fide PBs were readily distinguished from confounding CD138^+^ tubular epithelium by co-immunolabeling for CD38. Whole slide image analysis entailed the manual and automated exclusion of non-tissue whitespace, regions of tissue folding or other staining artifacts, and non-renal tissue from the analysis region of interest for each slide, followed by cell segmentation, and intensity threshold-based cell classification. LN tissue blocks typically included multiple needle tissue fragments per block, which were analyzed together to provide cumulative slide-level tissue cell enumeration. To explore the possibility that our bona fide PBs definition might miss a substantial population of recently transitioning PBs that had not yet begun to express CD138, as described in the peripheral circulation (3), we included a ‘permissively classified’ PB population that required only CD38 and Ki-67 immunolabeling. We recognized that this class could potentially be contaminated with non-PB CD38^+^/Ki-67^+^ cells, such as activated B and T cells, and natural killer (NK) cells (19).

### Quantitative spatial frequencies in TN and LN tissue

In this pilot cohort, enumerative image analysis revealed mean spatial densities of 6,314 B cells/mm^2^ in the TN controls and 249 B cells/mm^2^ in the LN biopsies (**Table 2**). Only 15.2% of B cells in TN and 2.5% in LN were classified as CD19^+^, highlighting that CD19 sensitivity within the context of this 5-plex assay was inadequate for enumerating B cell populations in LN tissue, where tertiary lymphoid structures are absent or rare. PCs had mean spatial densities of 565 PCs/mm^2^ in TN and 253 PCs/mm^2^ in LN. Interestingly, a substantial majority of these PCs were CD79a^+^, accounting for 88% and 72% of the PC populations in TN and LN, respectively. This indicates that the majority of these tissue PCs retain a B cell phenotype during maturation.

**Table 2:** Enumerated cells in tonsil and LN kidney tissues by marker-defined class.

Cell classes & marker status	Cumulative TN (N = 2) counts	Cumulative KD (N = 8) counts
**B cells (CD79a+/CD138-)**	**355850**	**3750**
B cells/mm^2^ tissue	6,314	249
B cells (% all cells)	45.0%	3.2%
CD19+ B cells (% all B cells)	15.2%	2.5%
**Plasma Cells (CD138+/CD38+/Ki-67-)**	**31831**	**3807**
plasma cells/mm^2^ tissue	565	253
plasma cells (% all cells)	4.0%	3.3%
CD79a+ plasma cells (% plasma cells)	88.04%	71.89%
**Permissively Classified Plasmablasts (CD38**+/**Ki-67**+)	**3812**	**282**
Permissive plasmablasts/mm^2^ tissue	67.6	18.7
Permissive plasmablasts (% all cells)	0.48%	0.24%
CD79a+ Permissive plasmablasts (% permissive plasmablasts)	73.6%	57.1%
**Bona fide CD138**+ **plasmablasts (CD138**+/**CD38+/Ki-67**+)	**3352**	**212**
Bona fide plasmablasts/mm^2^ tissue	59.48	14.09
Bona fide plasmablasts (% all cells)	0.42%	0.18%
Bona fide CD138+ plasmablasts (% permissive plasmablasts)	87.93%	75.18%
Bona fide CD138+ plasmablasts (% Aby-Secreting Cells)	9.53%	5.27%
CD79a+/CD138+ B. plasmablasts (% CD138+ B. plasmablasts)	83.68%	75.94%

**Figure 5:**
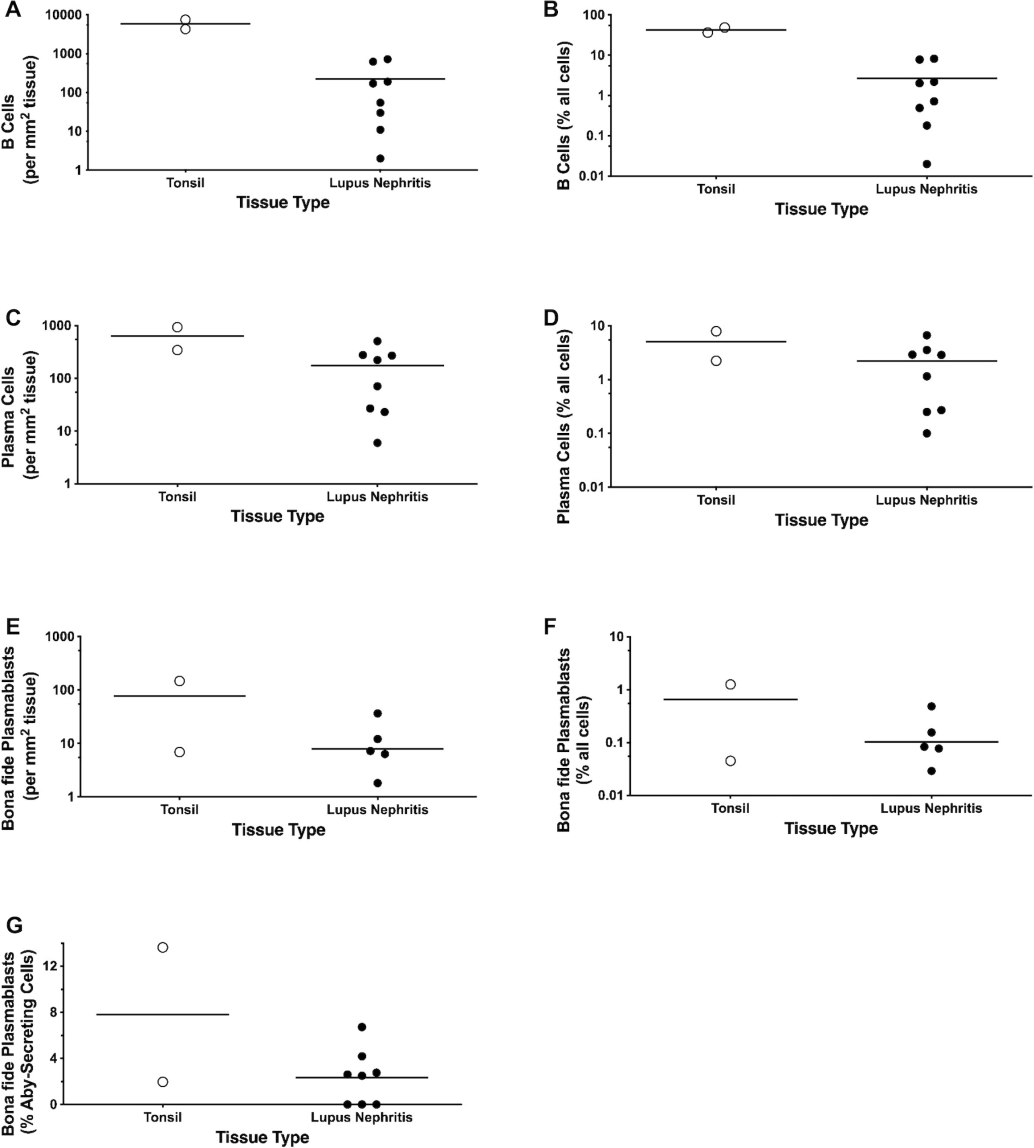
Individual slide analysis of 5-plex immunolabeling on two FFPE TN and eight LN sections. Spatial density [cells/mm2; **(A, C, E)**] and frequency [% of total cells: **(B, D, F)**] were generated for CD79a^+^/CD138^-^ B cells **(A, B)**, CD138^+^/CD38^+^/Ki-67^-^ PCs **(C, D)**, and bona fide CD138^+^/CD38^+^/Ki-67^+^ PBs **(E, F)**. **(G)** Proportion of bona fide PBs relative to total ASCs (PCs + PBs). Raw individual slide data is provided in **Supplementary Table 1**.

5-plex immunolabeling on two FFPE TN and eight LN sections was performed and indicated cell populations classified and enumerated as described in the Materials & Methods. Raw cumulative cell counts across all TN or LN tissue (bold entries) was tabulated to evaluate the mean frequency of each cell population within each tissue type (non-bold entries). Individual slide data is provided in **Supplementary Table 1** and **Figure 5**.

Bona fide PBs had mean spatial densities of 59 PBs/mm^2^ in TN and 14 PBs/mm^2^ in LN. This indicates that bona fide PBs accounted for ~5-10% of the total CD138^+^ antibody-secreting cell population in these tissues. Comparison of the two PB classification schemes showed that bona fide CD138^+^ PBs comprised a large majority of the permissive PB class, representing 88% and 75% of CD38^+^/Ki-67^+^ PBs in TN and LN, respectively. CD79a expression frequencies appeared to differ between the two PB classes in these samples: while bona fide PBs were highly CD79a^+^ (84% in TN and 76% in LN), permissively classified PBs were less often CD79a^+,^ accounting for only 74% and 57% in TN and LN, respectively. Notably, every CD79a^+^ cell within the permissively classified PB population was also a CD138^+^ bona fide PB across all tissues tested, confirming that the proliferative B-lineage ‘blast’ compartment in these tissues is uniformly CD138^+^.

## Discussion

The development and validation of this 5-plex IF and digital image analysis workflow provides a specific, fit-for-purpose methodology for evaluating B lineage cells in human FFPE tissue. This is particularly relevant for assessing the pharmacodynamic effects of B cell or PC depletion therapy, a strategy of increasing therapeutic interest in LN (20). This approach addresses a critical need in LN clinical trials, where needle biopsy material is scarce and availability of unstained slides for exploratory analysis often limited. By consolidating B cell and ASC enumeration onto a single slide, this assay maximizes data yield while preserving tissue for other critical diagnostic or exploratory analyses.

A primary technical challenge in the renal microenvironment is the endogenous expression of CD138 (Syndecan-1) on tubular epithelium, which can confound the identification of ASCs. We resolved this through the inclusion of CD38 as an obligate ASC co-marker. The requirement for dual positivity (CD138^+^/CD38^+)^ ensured the unambiguous classification of true ASCs, effectively segregating them from the CD138^+^/CD38^-^ tubular background. This gating strategy allows the workflow to resolve distinct B cell maturation stages, including infiltrating B cells, transitioning PBs, and differentiated PCs, while maintaining high specificity within the renal microenvironment. Furthermore, the inclusion of CD19 in this development panel allowed for a critical performance benchmark against CD79a. While CD19 is the canonical marker for flow cytometry and can be informative in a single-plex chromogenic assay in lymphoid tissue (17), our side-by-side comparison revealed that it lacks the sensitivity required to serve as a sole B cell marker in a multiplex IF format, especially in the renal interstitium where follicular lymphoid structures are infrequent. Thus, while CD19 remains informative for characterizing follicular structures and validating therapeutic target expression, CD79a establishes the requisite universal anchor for comprehensive B cell burden assessment in renal tissue using our methodology.

The comparative analysis of PB classification confirmed the necessity of including CD138 to ensure ASC specificity. The exploratory “permissive” PB definition (CD38^+/^Ki-67^+^ alone) showed significantly lower CD79a positivity (57% in LN) compared to bona fide CD138^+^ PBs (76% in LN). Notably, every CD79a^+^ cell within the ‘permissive’ PB population was also CD138^+.^ This suggests that CD138^-^ plasmablasts—early transitional cells often seen in blood—are effectively absent in these tissues. Consequently, the lower CD79a positivity rate in the permissive gate is likely driven by the inclusion of non-B-lineage cells, such as activated T and NK cells. Furthermore, this suggests that CD138 expression is acquired early in tissue differentiation, supporting CD138 as a comprehensive and sufficient marker for ASCs in the renal microenvironment.

The quantitative data derived from this workflow provides insight into the phenotypic composition and cellular density of the B lineage infiltrates in LN. The finding of comparable spatial densities of local B cells and PCs (mean of ~250 cells/mm² each) in the LN cohort, alongside far fewer proliferative PBs, underscores the kidney’s role not just as a site of inflammation, but as a niche of active, established antibody production. In the samples analyzed, this distribution pattern differed from the tonsil controls, where B cells outnumbered PCs (approximately 11:1), highlighting the distinction between the renal effector niche and secondary lymphoid tissue. Our data reveal a convergence of independent lines of evidence consistent with a model of *in situ* differentiation: B cells and ASCs frequently occupy shared perivascular and periglomerular neighborhoods (**Figures 4F–J**); quantitatively, their densities followed a similar trend across the cohort (**Supplementary Table 1**); and phenotypically, a substantial majority of both PCs (72% in LN) and bona fide PBs (76% in LN) retained CD79a expression (**Table 2**). Taken together, these findings are compatible with previous characterizations of the LN tubulointerstitium as an active site of adaptive immunity, where infiltrating B cells differentiate locally into antibody-secreting cells (21).

Methodologically, the CD79a finding has significant translational utility. Because renal tubular epithelium lacks CD79a expression, this high rate of retention identifies CD79a as a viable alternative lineage marker for distinguishing most ASCs from CD138^+^ tubular epithelium. Thus, in future study designs where multiplexing capacity is constrained or CD38 immunolabeling is technically compromised, a dual-positive CD79a^+^/CD138^+^ signature represents a validated, highly specific definition for renal PCs. This alternative gating strategy has recently been implemented to evaluate the pharmacodynamic impact in a clinical LN trial cohort (Manuscript in Preparation).

Beyond this technical utility, the biological interpretation of CD79a retention in ASCs has significant implications for LN pathogenesis. Our finding that the majority of tissue PCs retain CD79a aligns with recent descriptions of a human ‘B-cell program’ retention phenotype—a state characterized by the downregulation of CD20 but the continued capacity to present antigen to T cells and drive autoimmunity (22). While such populations are hypothesized to be long-lived and autonomous, the spatial and quantitative correlations we observed between B cells and ASCs (**Figure 4**, **Supplementary Table 1**) raise the alternative possibility of a local developmental axis that is more dynamic and renewing, as previously suggested in LN (2, 21). This dichotomy can be directly tested via longitudinal IF analysis following CD20-targeted deep B-cell depletion: if the long-lived phenotype predominates, these CD79a^+^ ASCs should persist as a resistant reservoir; conversely, if the compartment is dynamic and replenished by local differentiation of B cells, deep B cell depletion will erode this compartment by severing the upstream supply line. Our IF workflow provides the essential analytical framework to test these competing hypotheses by simultaneously quantifying the eradication of the B-cell precursor pool and the pharmacodynamic response of the downstream ASC compartment.

Our study has limitations inherent to its design as a methodological validation. First, the sample size of the LN pilot cohort (N = 8) was relatively small, intended primarily to capture a representative range of histological inflammation rather than to power a clinical correlation analysis; consequently, formal statistical hypothesis testing was not performed. While these observations regarding local differentiation align with published literature, they serve here as the foundational hypothesis for the large-scale deployment of this assay in the Phase III clinical trials. Second, due to the scarcity of clinical needle biopsy material, we could not perform orthogonal validation (e.g., flow cytometry) on matched tissue samples. However, our use of internal biological controls (tonsil) and the rigorous ‘leave-one-out’ validation strategy mitigates this risk. Third, regarding CD38 expression, our classification of CD79a^+^/CD138^-^/CD38^+^/Ki-67^-^ cells as B cells is intended to capture germinal center centrocytes and activated memory B cells (18). While this gate may arguably contain rare, quiescent CD138^-^ ‘circulating plasma cells’ described in blood (3), we prioritized specificity for defined proliferative plasmablast and tissue-resident plasma cell compartments to prevent overestimation of the ASC burden. This aligns with the known biology of plasma cell maturation, where CD138 acquisition is functionally linked to the establishment of residency within survival niches (14, 23), suggesting that CD138-negative blasts in the kidney are transient or negligible. Finally, this assay is strictly phenotypic; while the markers CD138, CD38, and CD79a robustly identify antibody-secreting cells, they do not directly measure autoantibody secretion rates. Future application of this workflow in interventional trials will be critical to linking these cellular densities to serological responses and clinical remission.

By defining rigorous phenotypic gates and overcoming specific histological challenges inherent to the renal microenvironment, this robust 5-plex IF workflow minimizes technical variability and ensures that changes in B-lineage cell density reflect true pharmacodynamic responses. This validated, fit-for-purpose methodology provides a high-precision tool for evaluating local tissue impacts of B-cell depletion therapy, deepening our understanding of disease pathogenesis and treatment responses.

## Data Availability

The original contributions presented in the study are included in the article/**Supplementary Material**. Further inquiries can be directed to the corresponding author.

## Supplementary material

Supplementary Figure 1 H&E verification of histopathology across the lupus nephritis biopsy cohort. **(A–H)** Representative high-magnification (40x) hematoxylin and eosin (H&E) images of diseased glomeruli from the eight LN biopsy specimens (LN 1–8). Panels A–H correspond to the individual cases detailed in **Supplementary Table 1**. These sections were stained and reviewed internally to independently verify vendor-provided clinical diagnoses according to ISN/RPS 2003/2018 criteria. The images demonstrate the defining pathological features used for classification, including endocapillary hypercellularity and crescent formation (Class III/IV) and glomerular basement membrane thickening (Class V). Scale bars = 100 µm.

Supplementary Figure 2 Intermediate-magnification visualization of 5-plex immunofluorescence across the LN cohort. **(A–H)** Representative multi-channel immunofluorescence images from the eight LN biopsy specimens stained with the optimized 5-plex panel (see **Supplementary Figure 3** for corresponding wide-field overviews). These images demonstrate consistent detection of glomerular and interstitial immune infiltrates across diverse tissue samples. Markers: CD79a (green), CD19 (magenta), Ki-67 (white), CD38 (yellow), CD138 (red), and DAPI nuclear counterstain (blue). Key structural features include glomerular tufts (gt) and vessels (v). Scale bars = 200 µm.

Supplementary Figure 3 Low-magnification overview of 5-plex immunofluorescence across the LN cohort. **(A–H)** Low-magnification wide-field immunofluorescence images of the same specimens featured in **Figure 4** and **Supplementary Figure 2**. These images enable a comprehensive view of the primary biopsy fragments, illustrating the global distribution of the 5-plex markers across entire biopsy fragments. Markers: CD79a (green), CD19 (magenta), Ki-67 (white), CD38 (yellow), CD138 (red), and DAPI nuclear counterstain (blue). Key structural features include glomerular tufts (gt) and vessels (v). Scale bars = 500 µm.

Supplementary Table 1 Individual slide analysis data. Individual slide analysis data of 5-plex immunolabeling on two FFPE TN and eight LN sections was performed as described in the Materials & Methods. Whole slide cell counts as a percentage of all tissue cells and as a spatial density per mm^2^ of tissue were generated for B cells, PCs, bona fide PBs, and permissively classified PBs. Bona fide PBs as a percentage of ASCs (PCs + PBs), and CD19 and CD79a marker status of selected cell populations, was also calculated. Selected individual slide data is also plotted in **Figure 5**.

Supplementary Table 2 optimized 5-plex immunofluorescence panel configuration.
