# Supplementary figures and images for: Resolving the renal microenvironment: a 5-plex immunofluorescence workflow to quantify B-lineage cells in FFPE lupus nephritis biopsies
Source: Front Immunol. 2026 Mar 25;17:1774536. doi: 10.3389/fimmu.2026.1774536 (PMC13058706; doi:10.3389/fimmu.2026.1774536)

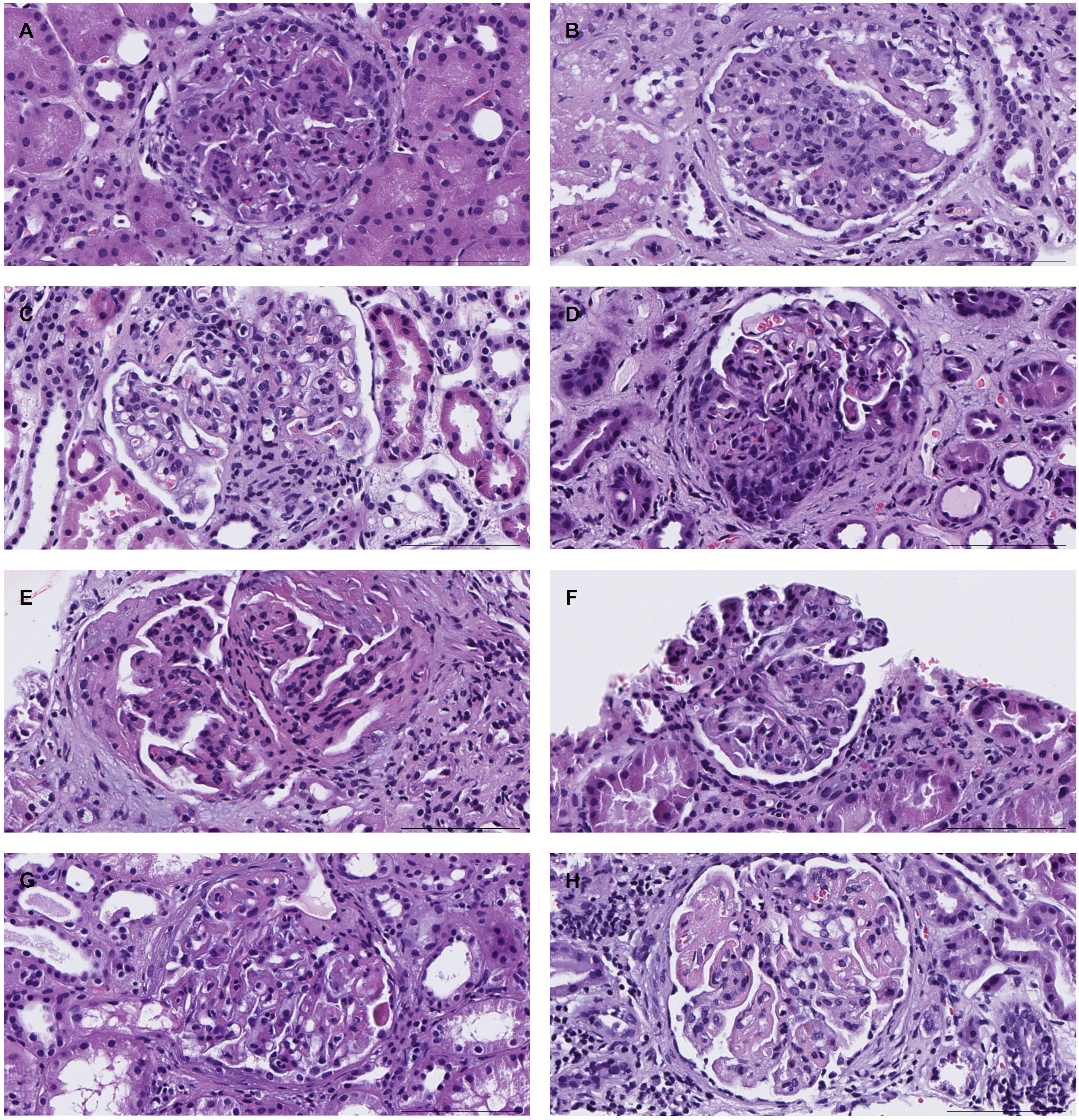

Supplement: Supplementary Figure 1 — H&E verification of histopathology across the lupus nephritis biopsy cohort. (A–H) Representative high-magnification (40x) hematoxylin and eosin (H&E) images of diseased glomeruli from the eight LN biopsy specimens (LN 1–8). Panels A–H correspond to the individual cases detailed in Supplementary Table 1. These sections were stained and reviewed internally to independently verify vendor-provided clinical diagnoses according to ISN/RPS 2003/2018 criteria. The images demonstrate the defining pathological features used for classification, including endocapillary hypercellularity and crescent formation (Class III/IV) and glomerular basement membrane thickening (Class V). Scale bars = 100 µm. [file Image1.jpeg]

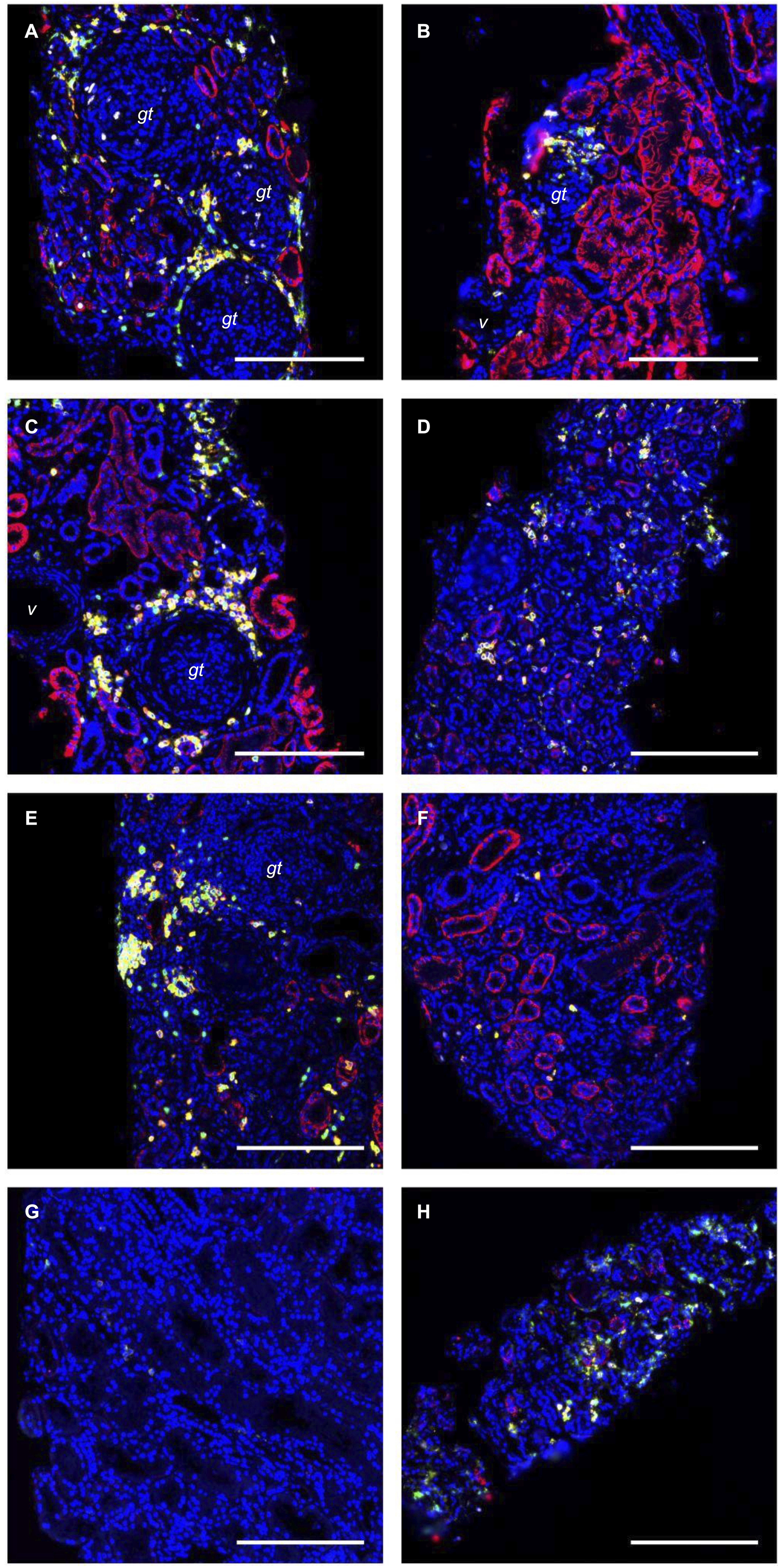

Supplement: Supplementary Figure 2 — Intermediate-magnification visualization of 5-plex immunofluorescence across the LN cohort. (A–H) Representative multi-channel immunofluorescence images from the eight LN biopsy specimens stained with the optimized 5-plex panel (see Supplementary Figure 3 for corresponding wide-field overviews). These images demonstrate consistent detection of glomerular and interstitial immune infiltrates across diverse tissue samples. Markers: CD79a (green), CD19 (magenta), Ki-67 (white), CD38 (yellow), CD138 (red), and DAPI nuclear counterstain (blue). Key structural features include glomerular tufts (gt) and vessels (v). Scale bars = 200 µm. [file Image2.jpeg]

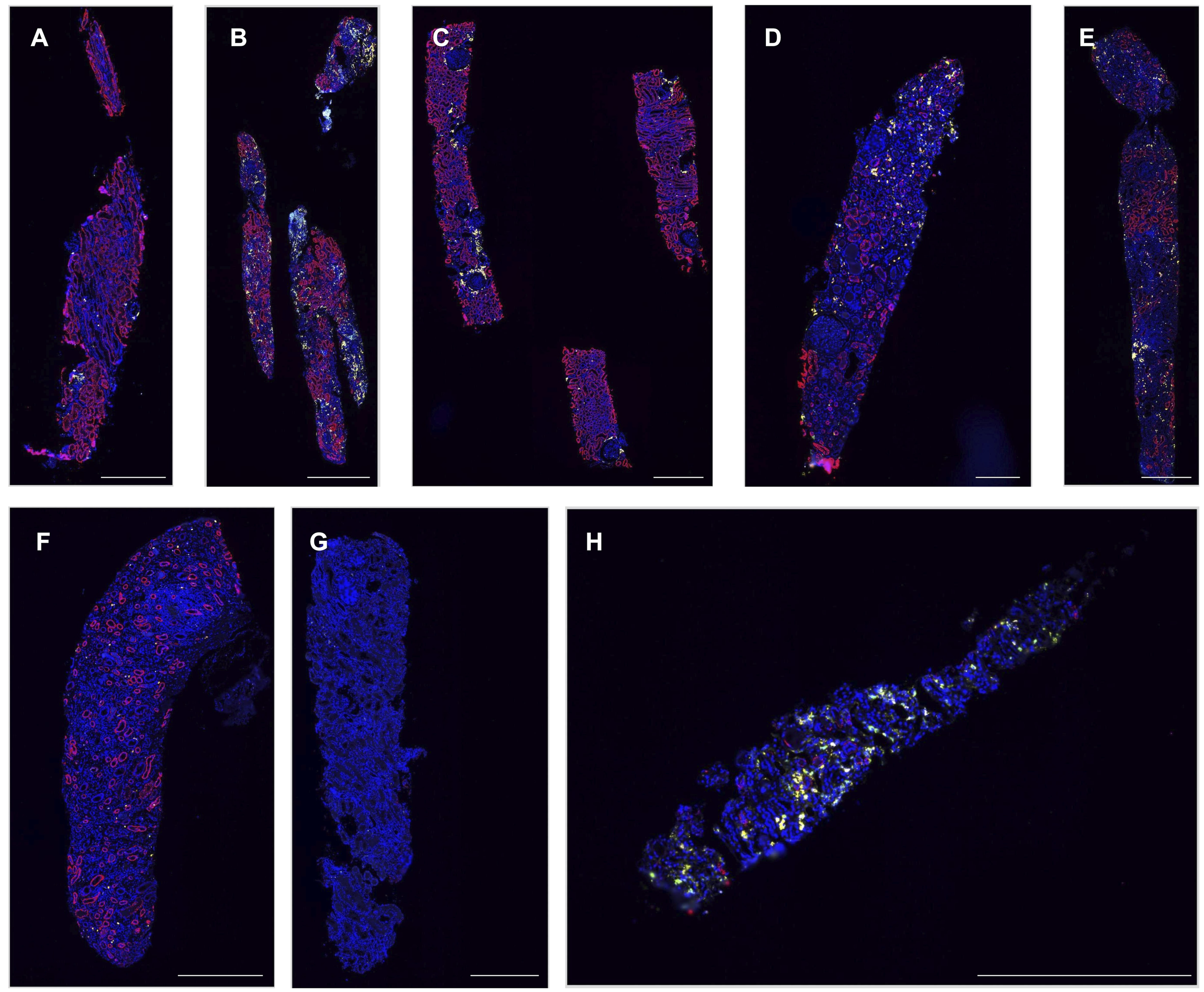

Supplement: Supplementary Figure 3 — Low-magnification overview of 5-plex immunofluorescence across the LN cohort. (A–H) Low-magnification wide-field immunofluorescence images of the same specimens featured in Figure 4 and Supplementary Figure 2. These images enable a comprehensive view of the primary biopsy fragments, illustrating the global distribution of the 5-plex markers across entire biopsy fragments. Markers: CD79a (green), CD19 (magenta), Ki-67 (white), CD38 (yellow), CD138 (red), and DAPI nuclear counterstain (blue). Key structural features include glomerular tufts (gt) and vessels (v). Scale bars = 500 µm. [file Image3.jpeg]
