# Supplementary material for: Resolving the renal microenvironment: a 5-plex immunofluorescence workflow to quantify B-lineage cells in FFPE lupus nephritis biopsies
Source: Front Immunol. 2026 Mar 25;17:1774536. doi: 10.3389/fimmu.2026.1774536 (PMC13058706; doi:10.3389/fimmu.2026.1774536)
Supplement: Supplementary Table 2 — optimized 5-plex immunofluorescence panel configuration. [file Table2.pdf]

Supplementary Table 2: Optimized 5-plex Immunofluorescence Panel Configuration

Sequence Position*	Marker	Clone	Host Species	Primary Concentration	Primary Incubation	Secondary Detection	Fluorophore	Fluorophore incubation
1	CD38	SPC32	Mouse	0.46 ug/ml	32 min, 37 ℃	OmniMap anti-Mouse HRP	Rhodamine 6G	8 mins, RT
2	CD79a	SP18	Rabbit	0.3 ug/ml	16 min, 37 ℃	OmniMap anti-Rabbit HRP	FAM	8 mins, RT
3	CD19	LE-CD19	Mouse	0.83 ug/ml	60 min, 37 ℃	OmniMap anti-Mouse HRP	Cy5	32 mins, RT
4	Ki-67	D3B5	Rabbit	0.3 ug/ml	60 min, 37 ℃	OmniMap anti-Rabbit HRP	Red 610	8 mins, RT
5	CD138	B-A38	Mouse	0.06 ug/ml	32 min, 37 ℃	OmniMap anti-Mouse HRP	Opal 780	see below†
Counterstain	Nuclei	N/A	N/A	N/A	N/A	N/A	DAPI	16 mins, RT

*Unstained slides were baked for 30 min at 70℃. Antigen retrieval prior to Sequence Position 1 was performed in Cell Conditioning 1 (CC1) for 64 min at 100℃. A blocking step in DISCOVERY Inhibitor (Neutralize) reagent was performed before every staining round for 8 min at RT. Secondary OmniMap-HRP detection was for 16 min at RT. Antibody elutions (CC2) were performed between each sequence position for 8 min at 100℃.

†CD138 detection utilized TSA-DIG amplification (1:400) for 8 min at RT, followed by CC2 elution for 8 min at 100℃, and visualization with Opal Polaris 780 (anti-DIG) at 1:25 for 32 min at RT.
